# Protein Denaturation Through the Use of Magnetic Molecularly Imprinted Polymer Nanoparticles

**DOI:** 10.3390/molecules26133980

**Published:** 2021-06-29

**Authors:** Charlotte Boitard, Aude Michel, Christine Ménager, Nébéwia Griffete

**Affiliations:** PHysico-Chimie des Electrolytes et Nanosystèmes InterfaciauX (PHENIX), Sorbonne Université, CNRS, 4 Place Jussieu, 75005 Paris, France; charlotte.boitard@orange.fr (C.B.); aude.michel@upmc.fr (A.M.)

**Keywords:** magnetic nanoparticles, molecularly imprinted polymer, magnetic hyperthermia, proteins, denaturation

## Abstract

The inhibition of the protein function for therapeutic applications remains challenging despite progress these past years. While the targeting application of molecularly imprinted polymer are in their infancy, no use was ever made of their magnetic hyperthermia properties to damage proteins when they are coupled to magnetic nanoparticles. Therefore, we have developed a facile and effective method to synthesize magnetic molecularly imprinted polymer nanoparticles using the green fluorescent protein (GFP) as the template, a bulk imprinting of proteins combined with a grafting approach onto maghemite nanoparticles. The hybrid material exhibits very high adsorption capacities and very strong affinity constants towards GFP. We show that the heat generated locally upon alternative magnetic field is responsible of the decrease of fluorescence intensity.

## 1. Introduction

Cell targeting as well as the inhibition of the protein function are still challenging nowadays. Conventional methods employed to target cells, such as aptamers [1,2], nanobodies [3] or chromobodies [4] have proved to be very effective and selective, but these biological molecules are difficult to produce, requiring either animal hosts [5,6] or time-consuming synthetic pathways [7]. For example, a strategy that is used in oncology consists on the targeting of growth factors (vascular endothelial growth factor or basic fibroblast growth factor), that are secreted by cancer cells, using small organic molecules [8,9], but identification of the possible inhibitors and determination of the best one are also really expensive and time-consuming [10]. Systems that are able to both target cancer specific proteins at the tumor place, if we accumulate them, inhibit the protein by modifying their three-dimensional configuration, easy to determine and fast to produce, could be formidable tools to overcome these limitations. 

Molecularly imprinted polymers (MIP), able to specifically bind molecules of interest only by knowing their structure, seem to perfectly fit these requirements. Indeed, they act as synthetic antibodies [11,12], because of the specific interactions between functional monomers and template protein, which will lead to the formation of the so-called imprints. They are tailor-made recognition sites, perfectly complementary to the target protein in terms of shape, size and functionality. Over the past few years, synthetic pathways were developed to obtain protein imprints [13] despite restrictions due to the fragile nature of these biomacromolecules, easily undergoing denaturation when submitted to the harsh conditions often used to imprint smaller molecules [14,15]. While MIP were initially mostly used for analytical or diagnosis purposes [16], interest focus nowadays on their use in nanomedicine, either to act as the targeting part of some drug delivery systems [17] or to inhibit the action of some proteins by making them inaccessible [18,19] or even for the reversible presentation of bioactivity and dynamic control of cell–material interactions [20].

Coupled more and more often to magnetic nanoparticles, e.g., maghemite γ-Fe_2_O_3_, the only use made of the new magnetic properties of MIP was to facilitate their manipulation, either during preparation steps [21,22,23] or for magnetic targeting [24]. Moreover, the application of an alternative magnetic field (AMF) of appropriate amplitude and frequency lead magnetic nanoparticles to release heat [25]. As magnetic heating is non-invasive, present no depth penetration limit and remote controllability, its use in nanomedicine is possible. Hence, an interesting field of research concerns now is the use of specific temperature profiles at the vicinity of magnetic nanoparticles for heating with minor to no macroscopic effect (hot spot effect) [26,27,28]. This local heating could be interesting in oncology after the accumulation of the magnetic MIP at the tumor place, using a magnet, and the adsorption of the interesting growth factors, then the application of AMF could led to the denaturation of the protein and to its inactive form. Hence the cancer cells will stop their growth. Even if the protein could be released from the magnetic MIP, as it is in its inactive form it cannot be recognized by the cancer cells. To the best of our knowledge, this local thermal effect was never employed to denature proteins adsorbed on imprinted polymers coupled to magnetic nanoparticles. 

We here present for the first time a novel concept for the inhibition of the protein function consisting on the use of magnetic molecularly imprinted polymer for the protein sequestration and the change of its chains structure using the heat dissipated by magnetic nanoparticles under AMF. We synthesized new magnetic protein imprinted polymers (labeled γ-Fe_2_O_3_@MIP) to target and denature green fluorescent protein (GFP). Attention was paid to the adsorption properties of γ-Fe_2_O_3_@MIP and the fate of adsorbed proteins. An AMF was successfully employed to take advantage of the magnetic hyperthermia properties of maghemite nanoparticles and denature the adsorbed proteins in bulk solution.

## 2. Results and Discussion

**Figure 1:**
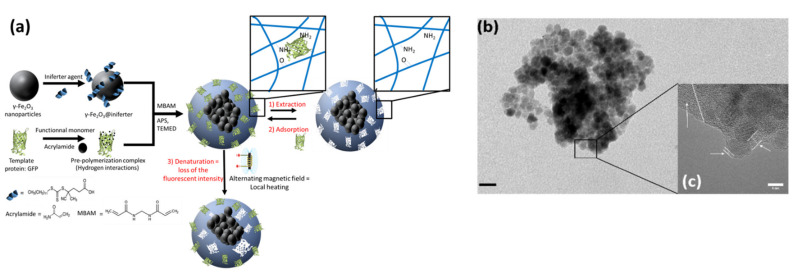
Preparation and characterization of magnetic GFP imprinted polymers γ-Fe_2_O_3_@MIP-GFP. (**a**): Scheme of the synthesis approach. Template protein: here GFP; MBAM: N,N-methylenebisacrylamide; APS: ammonium persulfate; TEMED: N,N,N′,N′-tetramethylethylenediamine. (**b**,**c**): Transmission electron microscopy of MIP-functionalized maghemite nanoparticles (**b**: scale bar 20 nm, **c**: scale bar: 6 nm). Polymer highlighted using white lines and arrows.

γ-Fe_2_O_3_@MIP-GFP nano-objects were synthesized in three steps, as displayed in Figure 1a [29].

**Figure 2:**
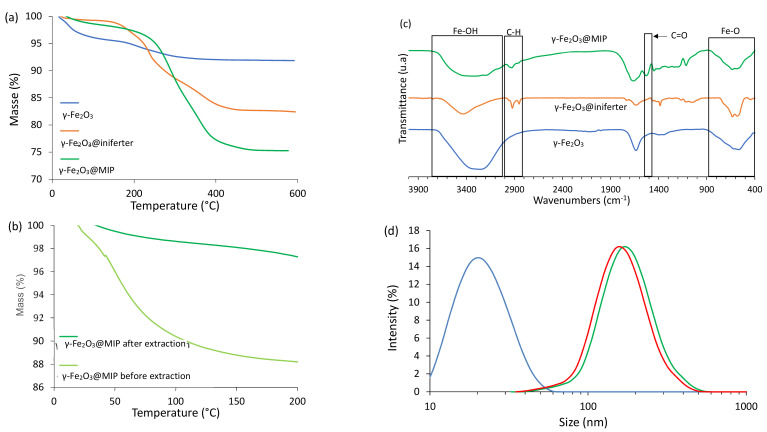
Characterizations of the different γ-Fe_2_O_3_ hybrid nano-objects. (**a**) TGA thermograms of bare, iniferter-functionalized and MIP functionalized maghemite nanoparticles. (**b**) TGA thermograms of γ-Fe_2_O_3_@MIP before and after protein extraction. (**c**) FTIR spectra of bare, iniferter-functionalized and MIP functionalized maghemite nanoparticles. (**d**): Dynamic light scattering profiles of bare (blue), MIP-functionalized γ-Fe_2_O_3_ nanoparticles (green) and NIP-functionalized γ-Fe_2_O_3_ nanoparticles (red).

After synthesis of magnetic nanoparticles using a co-precipitation method, they were functionalized with an initiation-transfer-termination (iniferter) agent [30] and mixed with pre-polymerization complexes composed of GFP and acrylamide. Polymerization was allowed to proceed at room temperature in water, and magnetic non-imprinted polymers (NIP) were synthesized the same way, without the GFP. Hybrid nano-objects were imaged using transmission electronic microscopy (TEM) as displayed in Figure 1b. One can see what seem to be only aggregates of magnetic nanoparticles, with a size ranging from 100 nm to 200 nm, coherent with the measured hydrodynamic diameter (197 ± 120 nm, Figure 2d). The presence of the imprints does not seem to affect the size of the final nano-objects, as MIP- and NIP-functionalized γ-Fe_2_O_3_ nanoparticles are very similar in size. High-resolution TEM evidenced an amorphous coating around these aggregates (see Figure 1c). Further analysis identified this amorphous coating as the polymer. Indeed, the size of the bare, MIP-functionalized and NIP-functionalized maghemite nanoparticles were determined using dynamic light scattering, as presented on Figure 2d. The presence of the polymer around the magnetic nanoparticles induces a strong increase in the size of the final objects. Fourier Transform infrared (FTIR) spectra of bare, iniferter-functionalized and polymer-coated maghemite nanoparticles were also recorded (Figure 2c). Bare maghemite nanoparticles display peaks at 628 cm^−1^ and 580 cm^−1^ corresponding to the Fe-O vibration, while FTIR spectrum of iniferter-functionalized nanoparticles depicts new peaks around 2900 cm^−1^ and 1630 cm^−1^, corresponding respectively to the stretch and scissoring movements of C-H bonds. Thus, surface functionalization of maghemite was effective. A new peak appears around 1530 cm^−1^ on the FTIR spectrum of γ-Fe_2_O_3_@MIP corresponding to C=O vibrations of acrylamide, and the peak previously situated around 1630 cm^−1^ shifted toward higher wavenumbers values, suggesting that polymer was successfully synthesized. Moreover, γ-Fe_2_O_3_@MIP displayed about 24% weight-loss, much higher than what was observed for bare or iniferter functionalized nanoparticles (8% and 18% respectively, Figure 2a), proving the existence of the organic part of the hybrid nanoparticles. γ-Fe_2_O_3_@MIP before and after protein extraction clearly show a 10% weight-loss difference at temperatures ranging from 100 to 200 °C (Figure 2b). This corresponds to the loss of proteins and confirms the presence of imprints as well as the effectiveness of the extraction method used.

**Figure 3:**
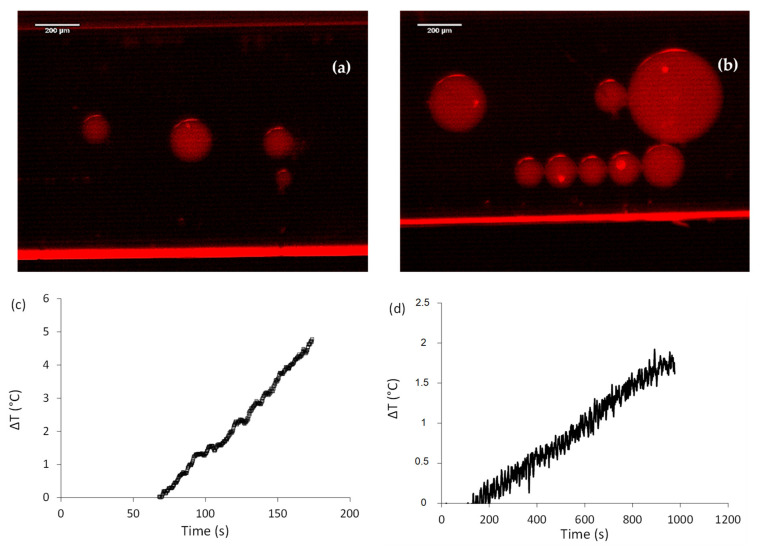
Magnetic characterizations of the different γ-Fe_2_O_3_ hybrid nano-objects. (**a**) Optic micrograph of a water-in-oil emulsion containing γ-Fe_2_O_3_@MIP before application of a magnetic field, scale bar: 200 µm. (**b**) Optic micrograph of a water-in-oil emulsion containing γ-Fe_2_O_3_@MIP after application of a magnetic field, scale bar: 200 µm. (**c**,**d**) Temperature increase of suspensions of bare (**c**) and MIP-functionalized (**d**) maghemite nanoparticles during the application of an alternating magnetic field at 335.1 kHz, 9 mT and 6 A, after 60 s of equilibration at room temperature.

Finally, in order to verify that maghemite nanoparticles remain magnetic while being coupled to the imprinted polymer, we sought to observe their response when submitted to a magnetic field. We prepared a water-in-oil emulsion, using egg-L-α-phosphatidylcholine (EPC) as surfactant. An aqueous suspension of fluorescent γ-Fe_2_O_3_@MIP (250 µL) was dispersed in 750 µL of chloroform containing 1% wt of EPC. After being well shaken, the emulsion was placed inside a capillary and observed using an optical microscope equipped a fluorescent source. The pictures presented in Figure 3a,b were taken before and after the application of a magnetic field, created using a neodymium-ferrite-bore magnet. We can observe in Figure 3a that before application of the magnetic field, the drops are rather well dispersed in the capillary, and distant from each other. As the entire of volume of the drops is fluorescent, the hybrid nano-objects are stable within it. After approaching the magnet, many drops were attracted in the observed area (see Figure 3b). We can also observe that the smallest drops aligned, forming a chain in the direction of the magnetic field. Thus, γ-Fe_2_O_3_@MIP nano-objects are able to respond to magnetic field, and polymer does not hinder the magnetism of the maghemite nanoparticles.

Magnetic properties were also assessed by measuring the temperature increase of dispersions containing bare or MIP-functionalized maghemite nanoparticles during the application of an alternating magnetic field at 335.1 kHz, 9 mT, 6 A. When submitted to such a magnetic field, magnetic nanoparticles will dissipate energy by producing heat, which will produce a localized temperature increase. The increase of temperature recorded for both dispersions, containing either maghemite nanoparticles (Figure 3c) or hybrid nano-objects (Figure 3d) prove that maghemite nanoparticles have magnetic hyperthermia properties. However, as the temperature increase is higher when submitted bare magnetic nanoparticles to AMF compared to magnetic MIP nanoparticles (at the same iron oxide concentration), we conclude that the local heating of the particles does not easily diffuse beyond the hybrid material and that the heating property is maintained by nanoparticles embedded inside an imprinted polymer. Even if nanoparticles aggregation inside the polymer induces a diminution of their specific adsorption rate from 10.5 W/g for bare nanoparticles to 6.2 W/g for hybrid nano-objects [31,32], these properties are maintained, even once embedded inside the imprinted polymer.

**Figure 4:**
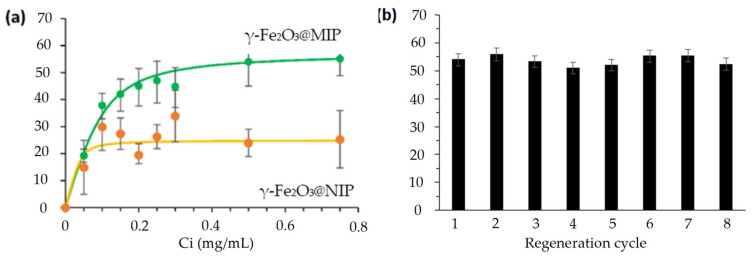
(**a**) Adsorption isotherms of GFP onto magnetic MIP and NIP, fitted with a Langmuir adsorption model, with V = 3 mL, m = 5 mg, Ci = 0–0.75 mg/mL, time: 2 h. (**b**) Influence of the number of adsorption-desorption cycles on the adsorption capacity of γ-Fe_2_O_3_@MIP-GFP. V = 3 mL, m = 5 mg and Ci = 0.5 mg/mL at room temperature.

The adsorption capacities of γ-Fe_2_O_3_@MIP and γ-Fe_2_O_3_@NIP were investigated by isothermal rebinding experiments. The adsorption capacities (Q) of both magnetic protein imprinted and non-imprinted polymers were determined. The adsorption curves displayed on Figure 4 show that the amount of adsorbed proteins increased with the initial concentration of GFP before reaching equilibrium. MIP has a maximal adsorption capacity of 57.5 mg/g, constant over eight adsorption-desorption cycles (Figure 4b) and adsorbs 2.28 times more GFP than the NIP. This confirms the existence of imprints and their efficiency to recognize the template protein.

To further investigate the protein-MIP interaction mechanism, a kinetic study was carried out. k_1_ is the pseudo-first order rate constant (min^−1^), k_2_ is the pseudo-second order rate constant (g/mg/min). Q_e_ and Q_t_ are the amount of protein adsorbed (mg/g) at equilibrium or at time t, respectively.

**Figure 5:**
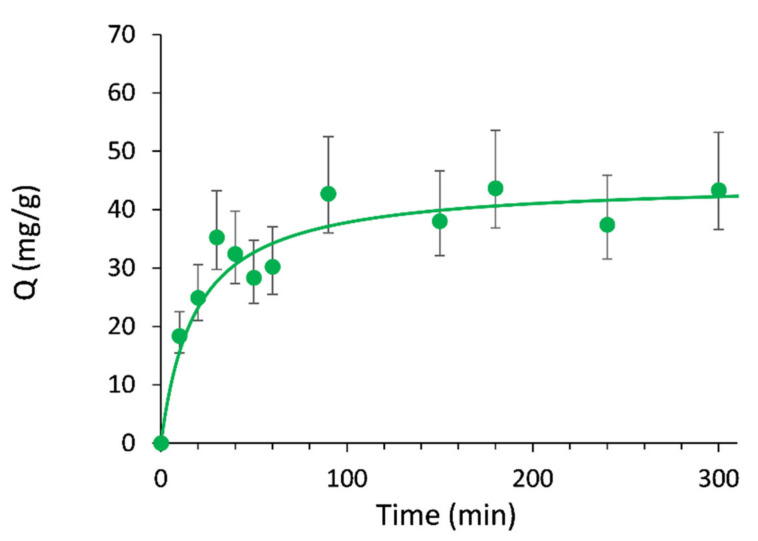
Adsorption kinetics of magnetic MIP toward GFP fitted with a pseudo-second order kinetic model, with V = 3 mL, m = 5 mg, C_i_ = 0.15 mg/mL, time: 0–5 h.

A pseudo second-order kinetic model was deemed suitable to fit the experimental data than a pseudo 1st order (Figure 5 as well as Table 1), suggesting that GFP adsorption on magnetic MIP obeys to a controlled diffusion process.

**Figure 6:**
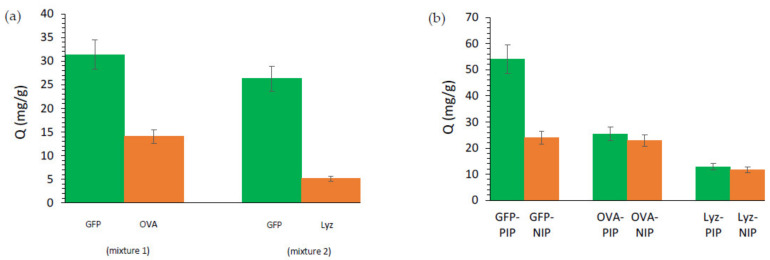
(**a**) Competitive adsorption tests performed on magnetic MIP with 5 mg of γ-Fe_2_O_3_@MIP dispersed in 3 mL of a double protein mixture solution, either GFP and OVA (mixture 1) or GFP and Lyz (mixture 2), each protein being present at a concentration of 0.5 mg/mL, and shaken at room temperature for 2 h. (**b**) Adsorption performances of γ-Fe_2_O_3_@MIP and NIP nano-objects. 5 mg of magnetic protein imprinted or non-imprinted polymer were added to 3 mL of HEPES buffer containing 0.5 mg/mL of reference proteins. The mixture was shaken at room temperature for two hours, to reach equilibrium.

Selective and competitive adsorption experiments were conducted using two non-fluorescent proteins with different isoelectric points and molecular weight (ovalbumin, OVA and lysozyme, Lyz) (Figure 6a). Magnetic GFP-imprinted polymers exhibit a higher recognition capacity toward GFP than OVA or Lyz, indicating that MIP have a much higher affinity for the template protein than for the competitive ones (Table S2). This may be due to the physical differences between them. OVA being bigger than GFP (Mw: 42.7 kDa against 27 kDa), it should have difficulties diffusing inside the polymer and entering the imprints. And at pH = 8, Lyz is charged positively while imprints were designed to interact with negatively charged proteins. Moreover, imprinting factors closed to 1 for both Lyz and OVA (Table S2) confirm that the recognition cavities of the imprinted polymers play no role in the adsorption of these competitive proteins. Non-specific interactions between amide functions of the polymer and proteins are sole responsible and γ-Fe_2_O_3_@MIP are selective toward GFP, a result confirmed by the good selectivity coefficients displayed (Figure 6b and Table S2). The lower adsorption capacities of the γ-Fe_2_O_3_@MIP nano-objects toward GFP in protein mixture 1 compared to the one single protein solution could be explained by a loss in the non-specific adsorption. Indeed, non-specific adsorption of Lyz or OVA will deprive the GFP of some non-specific adsorption sites.

We first looked at the passive desorption of GFP by dispersing 5 mg of γ-Fe_2_O_3_@MIP in 3 mL of a GFP solution at 0.8 mg/mL and shaken continuously at room temperature for two hours, to reach adsorption equilibrium. Then, particles were collected using a permanent magnet, supernatant was removed, and the GFP-saturated nanoparticles were dispersed in 3 mL of HEPES buffer (pH = 8, 200 mM). Sample was shaken continuously at room temperature and supernatant was collected using magnetic decantation and analysed by UV-Visible spectroscopy from time to time to determine the quantity of free protein. The amount of GFP desorbed by the magnetic imprinted polymers oscillates between 0% and 6% (mean value of 2.8%) of the maximal possible desorbable quantity, as displayed on Figure S1. Once GFP has been adsorbed by magnetic MIP, no significant passive desorption of trapped proteins occurs.

Then we investigated the effect of alternating magnetic field on the hybrid materials after GFP adsorption. The denaturation of GFP was investigated as follows, using the magnetic hyperthermia properties of maghemite nanoparticles. 5 mg of Fe_2_O_3_@MIP were first saturated with GFP as described above and dispersed in 3 mL of HEPES buffer (pH = 8, 200 mM). Sample was then placed in an Eppendorf tube inside the coil of a MagneTherm system, and an alternating magnetic field was applied for 5 to 45 min at different magnetic field (3.35 mT, 6.7 mT, 10 mT and 13.4 mT). Before and after application of the magnetic pulse, the magnetic suspension was directly analysed using fluorescence spectroscopy to determine the protein fluorescence loss (difference between the fluorescence before application and after AMF application). Protein denaturation experiment using alternating magnetic field was also carried out for a solution of GFP at 0.29 mg/mL in HEPES, pH = 8, 200 mM, without magnetic nanoparticles.

**Figure 7:**
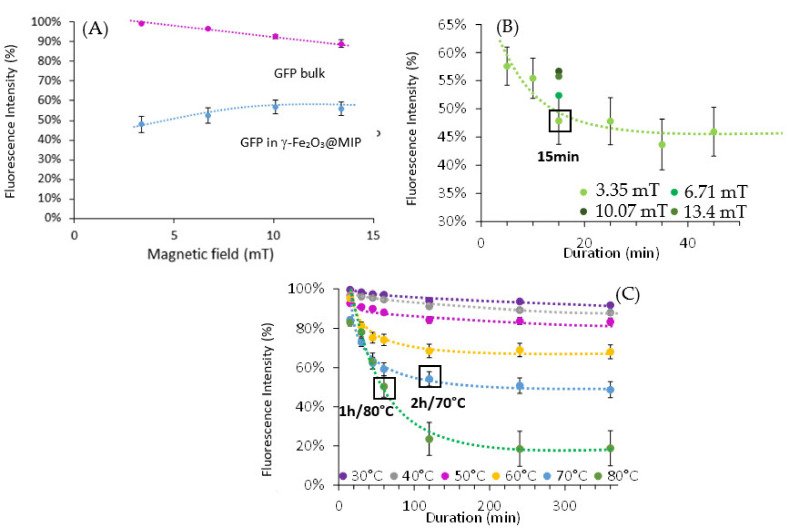
GFP denaturation experiments, with V = 3 mL, m = 5 mg ([γ-Fe_2_O_3_]: 8 mmol/L, (**A**) using magnetic hyperthermia (AMF at 335.1 kHz and various intensities) for 15 min, (**B**) in function of the time, (**C**) using different temperature and duration.

When we apply AMF on magnetic MIP containing GFP, the direct analysis of the fluorescent intensity decreases which is correlated to a modification of the GFP three-dimensional structure. Additionally, even if the magnetic field increases, the fluorescence loss of GFP is the same. This could be explained by the local heating temperature of the magnetic nanoparticles leading to the denaturation of the proteins that are near to the magnetic cores, and those who are far are not heated and not denatured. Moreover, the GFP alone in solution does not seem to undergo an as significant denaturation (labeled GFP bulk on Figure 7A), although it remains sensitive to the applied AMF. Thus, the fluorescence loss measured for proteins adsorbed by the hybrid nano-objects, and therefore their denaturation, seems to be due to the temperature increase induced by hyperthermia, and not only by the magnetic field itself.

Then, we looked at the duration effect of the AMF application on the GFP denaturation. As we can see on Figure 7B, even if we apply AMF during 45 min at 3.35 mT, the GFP fluorescence intensity lost does not exceed 60% obtained after 15 min of AMF application. As the results obtained with the magnetic field increase, the adsorbed GFP proteins not near to the magnetic nanoparticles cannot feel the locally temperature increase under AMF even if we increase the duration of AMF until 45 min.

The heat-triggered desorption of GFP was investigated as follows, using the magnetic hyperthermia properties of maghemite nanoparticles. 5 mg of Fe_2_O_3_@MIP were first saturated with GFP as described above and dispersed in 3 mL of HEPES buffer (pH = 8, 200 mM). Sample was then placed in an Eppendorf tube inside the coil of a MagneTherm system, and an alternating magnetic field was applied for 15 min at at 335.1 kHz, 9 mT and various intensities. Afterwards, supernatant was collected using magnetic decantation and analysed using UV-Visible spectroscopy to determine the quantity of desorbed protein. GFP-desorption after AMF application (Figure S1) at various intensities (in Amper) is slow (only 3% of GFP is desorbed whatever the intensity of AMF that is applied). The local temperature increase upon AMF should disrupt hydrogen bonding and should lead to protein desorption, but it seems that application of an AMF was not able to induce GFP desorption. This could be due to the big size of the protein that cannot diffuse inside the polymer pores existing in the MIP. As the desorption of the denatured protein does not occur, the effect of the adsorption of intact protein and the desorption of the denatured protein is limited because only small amount of adsorbed protein is inhibited and finally release. However, for in vivo applications of this protein denaturation method, to stop the proliferation of cancer cells for example, if the material is degraded in vivo at the tumor site, finally the denatured free protein won’t be recognized by the cancer cells and hence will limit the cancer cell propagation.

As any macroscopic temperature increase occurs when applying AMF, we decided to determine the temperature felt by the GFP when applying AMF leading to 45% of fluorescence loss after the application of AMF during 15 min at 3.35 mT. Hence, magnetic nanoparticles containing GFP were heated at temperature between 30 and 80 °C during 15 min to 6 h (Figure 7C). We can see that a significant fluorescent intensity loss appears if the heating is above 50 °C. If we compare the GFP fluorescence intensity when applying 15 min of AMF at 3.35 mT it seems to be equivalent to heating the particles at 70 °C during 2 h or at 80 °C during 1 h. When the particles heat upon AMF, the proteins near to the magnetic core are directly and quickly denatured. When the heating is performed, the time required to have the global good temperature for the protein denaturation may be longer. Moreover, in this case the denaturation occurs on all the proteins that is not the case when using AMF, apparently only the proteins near to the magnetic cores have a decrease of their fluorescent intensity and are inhibited. This could explain the difference obtained in the duration needed to have the equivalent loss of fluorescent upon AMF and when they are heated.

## 3. Materials and Methods

### 3.1. Materials

Ovalbumin (OVA), lysozyme (Lyz), acrylamide (AM), N,N-methylene-bis-acrylamide (MBAM), ammonium persulfate (APS), N,N,N’,N’-tertramethylethylenediamine (TEMED), 4-(2-hydroxyethyl)-1-piperazine-ethanesulfonic acid (HEPES), phosphate-buffered saline (PBS), 4-cyano-4-[(dodecylsulfanylthiocarbonyl)sulfanyl] pentanoic acid (iniferter agent), paraformaldehyde, Dulbecco’s modified Eagle’s medium with nutriment mixture F12 (DMEM/F12), fetal bovine serum (FBS), and PenStrep were provided by Sigma-Aldrich (Molsheim, France). Iron (II) chloride tetrahydrate (FeCl_2_·4H_2_O), iron (III) chloride hexahydrate (FeCl_3_·6H_2_O), iron (III) nitrate nonahydrate (Fe(NO_3_)_3_·9H_2_O), acetone, diethyl ether, ethanol at 96%, chloroform and methanol were provided by VWR Chemicals (Lutterworth, UK). Ammonia solution (NH_3_; 20%) and acetic acid were provided by Carlo Erba (Val-de-Reuil, France). Hydrochloric acid was provided by Merck (Molsheim, France). Acryloxyethylthiocarbamoyl-Rhodamine B was purchased from Polysciences, Inc. (Nanterre, France) The green fluorescent protein (GFP) was kindly provided to us by the Dahan group of the Curie Research Institute (Paris, France). All materials were used as received without any purification.

### 3.2. Characterization

Fourier Transform infrared (FTIR) spectra were recorded on a Tensor 27 spectrophotometer (Bruker, Palaiseau, France) in a KBr matrix. Thermogravimetric analysis (TGA) were carried out for bare maghemite nanoparticles and polymer coated nanoparticles using a SDT-Q600 system (TA Instruments, New Castle, DE, USA) under a nitrogen atmosphere with a heating rate of 10 °C/min up to 600 °C. Hydrodynamic diameters were measured at 25 °C using a Zetasizer Nano series (Malvern Instruments, Orsay, France). Images of the nano-objects were taken using an either a JEOL-100 CX transmission electron microscope (TEM) or a JEOL 2100F microscope (high resolution transmission electron microscopy, HRTEM) (JEOL, Croissy, France), and carbon-coated copper grids. UV-Vis adsorption spectra were recorded on an UVIKON XL spectrophotometer (SECOMAM, Champigny-sur-Marne, France). Measurements of protein fluorescence were performed using a Varian Cary Eclipse fluorescence spectrophotometer (Agilent, Agilent, France).

The specific adsorption rate (SAR) measurements of bare and MIP-functionalized maghemite nanoparticles and the adsorbed GFP denaturation experiments were carried out with a MagneTherm system (Nanotherics, Warrington, UK) equipped with a fluoroptic fiber thermometer. The sample was at room temperature before the application of an alternating magnetic field (335.1 kHz, 3.35–13.4, 5 or 15 min).

The SAR was calculated using Equation (1) after having fitted the experimental curves (temperature increase as a function of time) using the Box-Lucas equation presented as Equation (2):

(1) $$\mathrm{SAR}=A.\lambda .\frac{C_{p}}{C_{m}}$$

(2) $$\Delta T=A(1-e^{\lambda \left(t-t_{0}\right)})$$

where ΔT is the temperature variation, A and λ are experimental constants known as the Box-Lucas’ constants obtained by fitting the experimental curve, t_0_ is the moment the AMF was applied, C_m_ is the mass concentration of iron oxide nanoparticles (1.65% wt; polymer is neglected) and C_p_ is the heat capacity of the solution (4.12 J/g/K to consider both water and magnetic nanoparticles at the specified C_m_).

### 3.3. Synthesis of Functionalized Magnetic Nanoparticles

Maghemite nanoparticles were synthesized using a co-precipitation method as previously described by Massart [1]. Briefly, ferrous chloride (180 g) and ferric chloride (1.59 mol) were dissolved in 6% hydrochloric acid. Ammonia (1 L, 22.5%) was added to the mixture under vigorous magnetic stirring at room temperature. The reaction was allowed to proceed for 30 min. Then, the as-obtained magnetite was oxidized using ferric nitrate (323 g). The suspension was heated at 100 °C under magnetic stirring for 30 min. Maghemite nanoparticles were then washed three times with acetone and two times with diethyl ether, before being dispersed in water.

The surface of the nanoparticles was directly functionalized with an iniferter agent, using a protocol slightly modified from the one of Gonzato et al. [2]. In short, the iniferter agent, 4-cyano-4-[(dodecylsulfanylthiocarbonyl)sulfanyl]pentanoic acid (60 mg) was dissolved in ethanol (4 mL, 96%), followed by the addition of distilled water (26 mL) and γ-Fe_2_O_3_ nanoparticles (500 mg). The reaction was allowed to proceed at room temperature for 18 h under continuous orbital stirring. Then, functionalized nanoparticles were dialyzed using a 5/5 water/ethanol at 96% mixture until no more molecules were detected by conductivity measurements.

### 3.4. Synthesis of γ-Fe_2_O_3_@MIP

The synthesis of imprinted polymers was carried out as previously described [3]. Briefly, GFP (10 µmol) and acrylamide (30 mmol) were dissolved in HEPES buffer (150 mL, 200 mM, pH = 8). The mixture was allowed to react and form a pre-polymerization complex for 2 h at room temperature under constant magnetic stirring. Then, N,N-methylene-bis-acrylamide (3 mmol), acryloxyethylthiocarbamoyl-Rhodamine B (0.6 mmol), functionalized nanoparticles (300 mg) and APS (25 mg) were added and the mixture was nitrogen purged for 15 min under magnetic stirring. Lastly, TEMED (75 µL) was added to the mixture and the reaction was allowed to proceed for 18 h at room temperature under magnetic stirring. The final product was washed and template proteins were extracted using dialysis until no more fluorescence remains in the solution and no more proteins or molecules were detected by conductivity measurements. Dialysis baths were alternatively composed of a 5/4/1 water/methanol/acetic acid mixture and distilled water. Finally, particles were transferred into HEPES buffer (200 mM, pH = 8). Non-imprinted polymers (NIP) were synthetized using the same way but without the GFP as template.

### 3.5. In Vitro Adsorption Performances

The adsorption kinetics of γ-Fe_2_O_3_@MIP were investigated as follows: γ-Fe_2_O_3_@MIP (5 mg) were dispersed in a GFP solution (3 mL, 0.15 mg/mL) and shaken continuously at room temperature. Analyses were performed at certain pre-determined intervals, consisting in the collection of particles by an external magnetic field and analyse of supernatants using UV-visible spectroscopy and the excitation peak at 488 nm. The spectroscopy results allowed the assessment of the remaining concentration of protein and the determination of the quantity of adsorbed GFP, according to Equation (3):

(3) $$Q=\frac{\left(C_{i}-C_{f}\right)V}{m}$$

where C_i_ (mg/mL) and C_f_ (mg/mL) are respectively the initial and final concentrations of the protein samples, determined using UV-Visible spectroscopy, V (mL) is the volume of the protein solution and m (mg) is the mass of hybrid nano-objects in suspension.

The pseudo-first order kinetic model (Equation (4)) and the pseudo second-order kinetic model (Equation (5)) were used to fit the data.

(4) $$Q_{t}=Q_{e}\left[1-e^{-k_{1}t}\right]$$

(5) $$\frac{t}{Q_{t}}=\frac{t}{Q_{e}}+\frac{1}{k_{2}Q_{e}^{2}}$$

where k_1_ is the pseudo-first order rate constant (min^−1^]), k_2_ is the pseudo-second order rate constant (g/mg/min). Q_e_ and Q_t_ are the amount of protein adsorbed (mg/g) at equilibrium or at time t respectively.

The adsorption capacities (Q) of both magnetic protein imprinted and non-imprinted polymers were determined using the following protocol. γ-Fe_2_O_3_@MIP or NIP (5 mg) were dispersed in protein solutions (3 mL) at different initial concentrations. The resulting mixtures were shaken at room temperature for two hours. As previously described, particles were collected using an external magnetic field and supernatants were analysed using UV-Visible spectroscopy to determine the adsorption capacity according to Equation (3).

The adsorption of GFP on both γ-Fe_2_O_3_@MIP and γ-Fe_2_O_3_@NIP could be fitted using two different adsorption models. The first one is the Langmuir adsorption model (Equation (6)) and the second one is the Freundlich model (Equation (7)):

(6) $$\frac{C_{e}}{Q_{e}}=\frac{C_{e}}{Q_{\max}}+\frac{1}{K_{L}Q_{\max}}$$

(7) $$Q_{e}=K_{F}C_{e}^{1/m}$$

where C_e_ is the equilibrium concentration of GFP in solution (mg/mL), Q_e_ is the equilibrium amount of adsorbed GFP (mg/g), Q_max_ is the theoretical maximal amount of adsorbed GFP (mg/g), K_L_ is the Langmuir constant (mL/mg) related to the affinity of the adsorption sites, K_F_ ((mg/g)(mL/mg)^m^) is the Freundlich coefficient and m is the heterogeneity index.

OVA and Lyz were employed as reference proteins to determine the selectivity of γ-Fe_2_O_3_@MIP toward GFP. Magnetic protein imprinted or non-imprinted polymer (5 mg) were added to HEPES buffer (3 mL) containing reference proteins (0.5 mg/mL). The mixture was shaken at room temperature for two hours, to reach equilibrium. Then, particles were collected, supernatant was analysed using UV-Vis spectroscopy and the absorption peak at 290 nm for OVA and Lyz, and the quantity of adsorbed protein was determined using Equation (3).

To further investigate the selectivity of γ-Fe_2_O_3_@MIP toward GFP, competitive binding assays were carried out. Experiments were performed as follows: γ-Fe_2_O_3_@MIP (5 mg) were dispersed in a double protein mixture solution (3 mL), either GFP and OVA (mixture 1) or GFP and Lyz (mixture 2), each protein being present at a concentration of 0.5 mg/mL, and shaken at room temperature for 2 h. Then, particles were collected, and supernatants were analysed using UV-Vis spectroscopy. GFP remaining concentration was determined using the absorption peak at 488 nm, and contribution of GFP to the absorption peak at 290 nm was removed before determining the remaining concentration of the competitive protein. Quantities of adsorbed proteins were determined using Equation (3).

The imprinting factor (IF) and selectivity coefficient (SC) were calculated using Equations (8) and (9), were Q_MIP_ and Q_NIP_ (mg/g) are the adsorption capacities of magnetic protein imprinted or non-imprinted polymers toward GFP, and IF_t_ and IF_c_ are the imprinting factors for the template protein and the reference protein, respectively.

(8) $$\mathrm{IF}=\frac{Q_{\mathrm{MIP}}}{Q_{\mathrm{NIP}}}$$

(9) $$SC=\frac{{\mathrm{IF}}_{t}}{{\mathrm{IF}}_{c}}$$

All experiments were carried out in HEPES buffer solution at 200 mM and pH = 8.

### 3.6. Reusability of γ-Fe_2_O_3_@MIP

After having adsorbed GFP, γ-Fe_2_O_3_@MIP were eluted using alternatively a 9/1 methanol/acetic acid mixture and distilled water to remove proteins. After extraction, the regenerated γ-Fe_2_O_3_@MIP were reused for next adsorption of GFP.

### 3.7. Fate of Trapped Proteins

The passive desorption of GFP was investigated as follows: γ-Fe_2_O_3_@MIP (5 mg) were dispersed in a GFP solution (3 mL, 0.8 mg/mL) and shaken continuously at room temperature for two hours, to reach adsorption equilibrium. Then, particles were collected using a permanent magnet, supernatant was removed, and the GFP-saturated nanoparticles were dispersed in HEPES buffer (3 mL, pH = 8, 200 mM). Sample was shaken continuously at room temperature and supernatant was collected using magnetic decantation and analysed by UV-Visible spectroscopy from time to time to determine the quantity of free protein.

The heat-triggered desorption and denaturation of GFP was investigated as follows, using the magnetic hyperthermia properties of maghemite nanoparticles. γ-Fe_2_O_3_@MIP (5 mg) were first saturated with GFP as described above, and dispersed in HEPES buffer (3 mL, pH = 8, 200 mM). The sample was then placed in an Eppendorf tube inside the coil of a MagneTherm system, and an alternating magnetic field was applied for 15 min (335.1 kHz). After application of the magnetic pulse, suspension was analysed using fluorescence spectroscopy to determine the protein fluorescence loss. Protein denaturation experiment using alternating magnetic field was also carried out for a solution of GFP (0.29 mg/mL) in HEPES (pH = 8, 200 mM), without magnetic nanoparticles.

Afterwards, supernatant was collected using magnetic decantation and analysed using UV-Visible spectroscopy to determine the quantity of desorbed protein. As heating could denature the GFP and modify its fluorescence properties, we did not use the absorption peak at 488 nm, but the one at 285 nm for UV-visible spectroscopy measurements.

## 4. Conclusions

In conclusion, we have developed a new tool to specifically target proteins by means of magnetic molecularly imprinted polymers, prepared using iniferter-functionalized maghemite nanoparticles and polyacrylamide. Evaluation of the adsorption performances showed that γ-Fe_2_O_3_@MIP displays relatively fast adsorption kinetics, good specificity and selectivity, and great recyclability. Polymers exhibit a great affinity toward GFP, as no significant passive or heat-induced desorption occurred. Finally, γ-Fe_2_O_3_@MIP were employed to successfully denature targeted proteins, displaying a great potential for medical applications and the inhibition of the protein function.

## Figures and Tables

**Table 1:** Parameters of pseudo-first order and pseudo-second order adsorption kinetics models for GFP on γ-Fe_2_O_3_@MIP.

Adsorption Model	R	Q_e,theo_ (mg/g)	Q_e,exp_ (mg/g)	k
Pseudo 1st order	0.853	40.2	44.3	4.5 × 10^−2^ min^−1^
Pseudo 2nd order	0.978	44.8	44.3	1.19 × 10^−3^ g/mg/min

## Supplementary Materials

The following are available online, Table S1: Parameters of isotherm models for GFP adsorption on both γ-Fe_2_O_3_@MIP and γ-Fe_2_O_3_@NIP, Table S2: Recognition selectivity of magnetic imprinted and non-imprinted nano-objects toward different proteins, Figure S1: GFP-desorption after heating of protein-saturated γ-Fe_2_O_3_@MIP nano-objects for 15 min using magnetic hyperthermia (alternating magnetic field at 335.1 kHz, 9 mT and various intensities).
