# Supplementary material for: Protein Denaturation Through the Use of Magnetic Molecularly Imprinted Polymer Nanoparticles
Source: Molecules. 2021 Jun 29;26(13):3980. doi: 10.3390/molecules26133980 (PMC8272029; doi:10.3390/molecules26133980)
Supplement: Supplementary file 1 [file molecules-26-03980-s001.zip › molecules-1265164-supplementary.pdf]

# Protein denaturation through the use of magnetic molecularly imprinted polymer nanoparticles

Charlotte Boitard, Aude Michel-Tourgis, Christine Ménager*, Nébéwia Griffete*

Sorbonne Université, CNRS, PHysico-chimie des Electrolytes et Nanosystèmes InterfaciauX, PHENIX, F-75005 Paris, France.

nebewia.griffete@sorbonne-universite.fr

christine.menager@sorbonne-universite.fr

| Sample and adsorption model | $Q_{max,exp}$ (mg/g) | R | $Q_{max,theo}$ (mg/g) | K | m |
|---|---|---|---|---|---|
| γ-$Fe_2O_3$@MIP, Langmuir | 57.5 | 0.999 | 57 | $1.14x10^6$ $M^{-1}$ | - |
| γ-$Fe_2O_3$@NIP, Langmuir | 24.9 | 0.991 | 25 | $6.56x10^6$ $M^{-1}$ | - |
| γ-$Fe_2O_3$@MIP, Freundlich | 57.5 | 0.995 | 60 | $1x10^{13}$ $(mg/g)(mL/mg)^{0,13}$ | 0.13 |
| γ-$Fe_2O_3$@NIP, Freundlich | 24.9 | 0.489 | 29 | $5.28x10^{16}$ $(mg/g)(mL/mg)^{0,09}$ | 0.09 |

*Table S1. Parameters of isotherm models for GFP adsorption on both γ-$Fe_2O_3$@MIP and γ-$Fe_2O_3$@NIP.*

| Protein | IF | SC |
|---|---|---|
| GFP | 2.28 | / |
| OVA | 1.11 | 2.05 |
| Lyz | 1.10 | 2.07 |

*Table S2. Recognition selectivity of magnetic imprinted and non-imprinted nano-objects toward different proteins.*

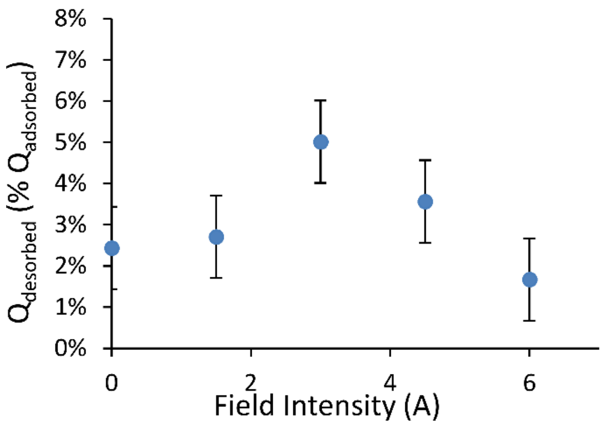


*Figure S1. GFP-desorption after heating of protein-saturated γ-$Fe_2O_3$@MIP nano-objects for 15 min using magnetic hyperthermia (alternating magnetic field at 335.1 kHz, 9 mT and various intensities).*
